# Atypical clinical presentation of a subset of patients with anti-RNA polymerase III - non-scleroderma cases associated with dominant RNA polymerase I reactivity and nucleolar staining

**DOI:** 10.1186/ar3422

**Published:** 2011-07-22

**Authors:** Angela Ceribelli, Malgorzata E Krzyszczak, Yi Li, Steven J Ross, Jason YF Chan, Edward KL Chan, Rufus W Burlingame, Tyler T Webb, Michael R Bubb, Eric S Sobel, Westley H Reeves, Minoru Satoh

**Affiliations:** 1 Department of Oral Biology, University of Florida, 1395 Center Drive, Gainesville, FL 32610-0424, USA; 2 Division of Rheumatology and Clinical Immunology, Department of Medicine, University of Florida, 1600 SW Archer Road, Gainesville, FL 32610-0221, USA; 3 INOVA Diagnostics, Inc., 9900 Old Grove Road, San Diego, CA, 92131-1638, USA; 4 Department of Pathology, Immunology, and Laboratory Medicine, University of Florida, 1600 SW Archer Road, Gainesville, FL 32610-0221, USA

## Abstract

**Introduction:**

Anti-RNA polymerase III (RNAP III) antibodies are highly specific markers of scleroderma (systemic sclerosis, SSc) and associated with a rapidly progressing subset of SSc. The clinical presentation of anti-RNAP III positive patients, onset of Raynaud's phenomenon (RP) and SSc in unselected patients in a rheumatology clinic were evaluated.

**Methods:**

Autoantibodies in sera from 1,966 unselected patients (including 434 systemic lupus erythematosus (SLE), 119 SSc, 85 polymyositis/dermatomyositis (PM/DM)) in a rheumatology clinic were screened by radioimmunoprecipitation. Anti-RNAP III positive sera were also tested by immunofluorescence antinuclear antibodies and anti-RNAP III ELISA. Medical records of anti-RNAP III positive patients were reviewed.

**Results:**

Among 21 anti-RNAP III positive patients, 16 met the American College of Rheumatology (ACR) SSc criteria at the initial visit but 5 did not; diagnoses were vasculitis, early polyarthritis, renal failure with RP, interstitial lung disease, and Sjögren's syndrome. The first two patients developed rapidly progressive diffuse SSc. An additional case presented with diffuse scleroderma without RP and RP developed two years later. Anti-RNAP III antibodies in these 6 cases of atypical clinical presentation were compared with those in 15 cases of typical (SSc with RP) cases. Anti-RNAP III levels by ELISA were lower in the former group (*P *= 0.04 by Mann-Whitney test) and 3 of 6 were negative versus only 1 of 15 negative in the latter (*P *< 0.05 by Fisher's exact test). Three cases of non-SSc anti-RNAP III positive patients had predominant reactivity with RNAP I with weak RNAP III reactivity and had a strong nucleolar staining. Three anti-RNAP III patients, who did not have RP at the initial visit, developed RP months later. Scleroderma developed prior to RP in 5 out of 16 (31%) in the anti-RNAP III group, but this was rare in patients with other autoantibodies. The interval between the onset of RP to scleroderma was short in anti-RNAP III positive patients.

**Conclusions:**

Anti-RNAP III antibodies are highly specific for SSc; however, a subset of anti-RNAP III positive patients do not present as typical SSc. The interval between RP and scleroderma in this group is short, and 31% of patients developed scleroderma prior to RP in this group. Anti-RNAP III positive patients may not present as typical SSc and detecting anti-RNAP III may have predictive value.

## Introduction

Specific autoantibodies in systemic rheumatic diseases are useful biomarkers associated with certain diagnoses and/or clinical manifestations [1]. Several autoantibodies, including anti-topoisomerase I (topo I), -centromere (ACA), -RNA polymerase III (RNAP III), -U3RNP/fibrillarin, and -Th/To, have been reported to be associated with scleroderma (systemic sclerosis, SSc); some are considered highly specific disease markers while others are considered relatively specific [2]. Anti-RNAP III that is considered highly specific for SSc, is a relatively new disease marker of SSc; however, it has become a popular test in the last several years thanks to the wide availability of commercial ELISA kits [1,2]. Detecting anti-RNAP III in some undiagnosed patients would not be totally unexpected, considering that autoantibodies are usually produced prior to typical clinical manifestations [3]. However, detection of anti-RNAP III in non-SSc patients or prior to clinical SSc has rarely been reported [4]. Although anti-RNAP III antibodies are associated with rapid progression of the disease and the interval between the onset of Raynaud's phenomenon (RP) and SSc is short [2,5], the time course of the onset of RP and SSc has not been well described. In the present study, the clinical features of anti-RNAP III positive patients in a cohort of an unselected population in a rheumatology clinic that includes undiagnosed patients and patients with a wide variety of diagnosis, were characterized. The relationships among detection of anti-RNAP III antibodies, onset of RP, and development of sclerodermatous skin changes, were also systematically analyzed.

## Materials and methods

### Patients

All 1,966 subjects enrolled in the University of Florida Center for Autoimmune Diseases (UFCAD) registry from 2000 to 2010 were studied. Diagnoses of the patients include 434 SLE, 119 SSc, 85 polymyositis/dermatomyositis, and various other diagnoses, and many remained undiagnosed for a specific systemic autoimmune disease. At each visit of the enrolled subjects, a form with a standard check list of symptoms and physical findings, including Raynaud's phenomenon and sclerodermatous skin changes, was filled out by physicians in addition to an entry in the medical chart. The data from the form were then entered into a computer database. Clinical information for the study was from the database and chart records. Raynaud's phenomenon was defined as sudden reversible white pallor of acral structures, which typically is followed by color changes to purple then to red [6]. The protocol was approved by the Institutional Review Board (IRB). This study meets and is in compliance with all ethical standards in medicine. Informed consent, including the publication of the study, was obtained from all patients according to the Declaration of Helsinki.

### Immunoprecipitation

Autoantibodies in sera from the initial visit of each patient were screened by immunoprecipitation (IP) using ^35^S-methionine labeled K562 cell extract. Anti-RNAP III were determined using reference sera [4]. Specificity of autoantibodies was determined using previously described reference sera [7].

### Immunofluorescent antinuclear antibodies

Immunofluorescent antinuclear/cytoplasmic antibodies (HEp-2 ANA slides; INOVA Diagnostics, San Diego, CA, USA) were tested using a 1:80-diluted human serum and DyLight488 donkey IgG F(ab')_2 _anti-human IgG (gamma-chain specific, 1:200 dilution; Jackson ImmunoResearch Laboratories, Inc., West Grove, PA, USA) [8].

### ELISA

Sera were tested for IgG anti-RNAP III antibodies using a commercial ELISA kit (QUANTA Lite^® ^RNA Pol III, INOVA Diagnostics) following the manufacturer's instruction.

### Statistical analysis

Data between groups were compared by the Mann-Whitney test using Prism 5.0 for Macintosh (GraphPad Software, Inc., San Diego, CA, USA). *P *< 0.05 was considered significant.

## Results

Autoantibodies to RNA polymerase I/III were found in 21 patients (1.1% of 1,966); 18 Caucasians, 2 African Americans, and 1 of mixed ethnic background. Sixteen of 21 cases had a diagnosis of SSc at the initial visit while 5 did not (Table 1). In the Caucasian patients, 14 out of 18 were diagnosed as having SSc at the initial visit. Four patients (cases 1, 3, 4, 5) did not fulfill the SSc criteria at the initial visit when serum anti-RNAP I/III antibodies were detected. Two African American patients had a diagnosis of SSc at the initial visit. A patient of mixed ethnic background did not meet the American College of Rheumatology (ACR) criteria at the initial visit (case 2). Patients who did not meet SSc criteria at the initial evaluation are summarized below and in Table 1. A brief history of an additional case 6, in which sclerodermatous change was followed by RP two years later, is also described.

**Table 1:** Five anti-RNAP III positive cases that were not classified as SSc at their initial visit

	1		2		3	4	5
	Initial	f/u	Initial	f/u			
Diagnosis	Vasculitis?	SSc	Poly -arthritis	SSc	Sine SSc?	ILD	Sjögren's syndrome
Race/gender	WF		Mixed F		WF	WF	WF
Anti-RNAP III ELISA (u)	16	NA	99	127	8	15 to 47	42
Proximal scleroderma	N	Y < 10 mo	N	Y 3 mo	N	N	N
Sclerodactyly	N	Y < 10 mo	N	Y 2 mo	N	N	N
Pitting scar	N	N	N	N	N	N	N
ILD	N	N	N	N	N	Y	N
Raynaud's phenomenon	N	?	N	Y 6 mo	Y For 10 y	N	N
Other		Renal crisis 10 mo		Flex.cont PH	ARF (TMA), DCM		

ARF, acute renal failure; DCM, dilated cardiomyopathy; W, white; F, female; Flex cont, flexion contracture; f/u, follow up; ILD, interstitial lung disease; N, no; NA, not available; PH, pulmonary hypertension; SSc, systemic sclerosis, scleroderma; TMA, thrombotic microangiopathy; Y, yes

Case 1: A fifty-year-old female was hospitalized for shortness of breath and chest pain in March 2000. Numbness in her left second digit also developed and became progressively ischemic and painful, resulting in amputation due to gangrene in April of that year. A hypercoagulable state secondary to malignancy was suspected, but nothing was found. In May 2000, she developed ischemic areas on the tips of the fingers and toes, and was put on anticoagulation therapy. Her ANA (speckled pattern, titer 1:640) and anti-RNAP I/III antibodies were positive but no scleroderma or RP was noted. Prednisolone (40 mg/day) was started and her condition was followed at her local clinic. She developed proximal scleroderma and scleroderma renal crisis in March 2001.

Case 2: A 39-year-old female developed polyarthritis involving the metacarpophalangeal joints (MCPs), proximal interphalangeal joints (PIPs), wrists and ankles in August 2004. The initial diagnosis was early synovitis without evidence of systemic rheumatic disease. Rheumatoid factor (RF) and anti-CCP were negative but ANA (speckled pattern, titer 1:160) and anti-RNAP I/III antibodies were positive. She developed sclerodermatous changes in her fingers, forearms and face in October 2004, which rapidly progressed to proximal scleroderma in November. Monthly i.v cyclophosphamide therapy was started, followed by prednisolone (50 mg/day) in December. She started having RP in February 2005 and verapamil and bosentan were started. Right heart catheterization in March 2005 suggested mild pulmonary hypertension (PH). Scleroderma progressed to involve the chest, shoulders and abdomen, and flexion contractures of the fingers were noted in March 2005. She was hospitalized in 2008 for anemia caused by gastric antral vascular ectasia (GAVE).

Another very similar case was seen. A 32-year-old Caucasian female was classified as having early synovitis (wrists, MCP, PIP joints) with a positive ANA (speckled and nucleolar pattern). A year later she developed proximal scleroderma and visited the UFCAD. RP developed six months later.

Case 3: A 57-year-old female, who had a 10-year history of RP, visited a clinic for worsening RP in April 2002, when hydroxychroloquine was started. In May 2002 she was hospitalized elsewhere for dyspnea, hypoxia and a nonproductive cough. Pleural effusions and heart failure were found, and her cardiac ejection fraction was 25%. A diagnosis of dilated cardiomyopathy was made. She also developed hypertension and renal dysfunction. Prednisolone (60 mg/day) and azathioprine were started but the latter was discontinued due to a rash. In July 2002, she developed renal failure and hemodialysis was started. Her kidney biopsy revealed thrombotic microangiopathy. Decreased sensation of her lower left leg was diagnosed as neuropathy. She visited UFCAD in July 2002. No sclerodactyly was noted but one teleangiectasia was found in the digit. ANA (speckled and nucleolar pattern, titer 1:80) and anti-RNAP I/III were positive.

Case 4: A 67-year-old female who was followed by a pulmonologist for a three-year history of respiratory symptoms was referred to the UFCAD for a positive ANA (speckled and nucleolar) and ILD. Anti-RNAP I/III antibodies were positive. She did not have rheumatological symptoms, including arthritis, sclerodermatous changes, or RP at the one-year follow-up.

**Figure 1:**
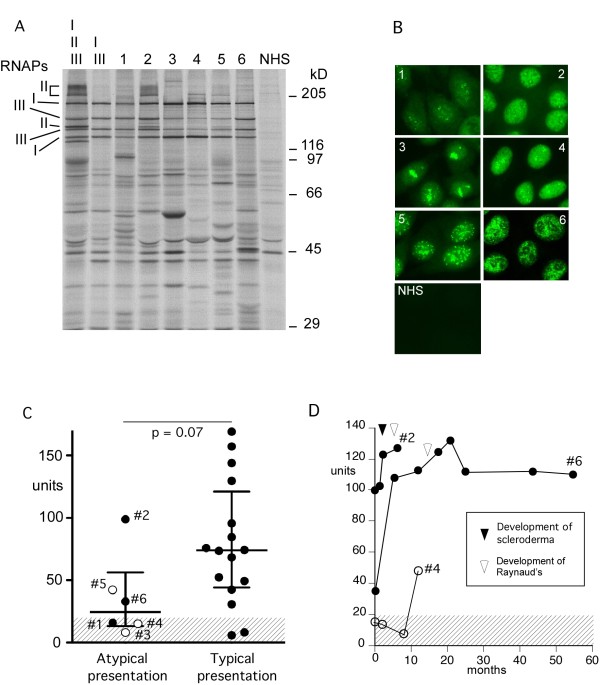
**Anti-RNAP III antibodies by immunoprecipitation, immunofluorescence, and ELISA**. **A**). Immunoprecipitation using ^35^S-methionine labeled K562 cell extract. ^35^S-methionine labeled K562 cell extract was immunoprecipitated by serum samples from patients with anti-RNA polymerase I/III, who had atypical clinical presentations (Table 1) and controls. RNAPs, RNA polymerases; I, II, III, two largest subunits of RNA polymerase I, II, and III, respectively; lane I, II, III, a reference serum with anti-RNAP I/II/III; lane I, III, a reference serum with anti-RNAP I/III; 1 to 6, IP with sera from cases 1 to 6; NHS, normal human serum; positions of molecular weight markers are also indicated. **B**). Immunofluorescence. HEp-2 ANA slide was stained with sera from cases 1 to 6 (Table 1) at 1:80 dilutions. **C**). Anti-RNAP III levels by ELISA. Sera from 21 cases with anti-RNAP I/III were tested by ELISA. Six cases of atypical presentation (Table 1) and 15 cases of typical presentation of SSc are shown. *P *= 0.04 by Mann-Whitney; closed circle, SSc; open circles, atypical cases in Table 1. **D**). Anti-RNAP III levels over time. Sera from cases 2, 4 and 6 over time were tested by anti-RNAP III ELISA. Time points of onset of sclerodermatous skin change (black arrowhead) or Raynaud's phenomenon (white arrowhead) are indicated. A cut-off (20 units) is shown as shaded area.

Case 5: A 68-year-old female was followed for Sjögren's syndrome. She had dry eyes, a dry mouth and a positive salivary gland biopsy but no sclerodermatous skin changes, RP or ILD. She was also positive for ACA by immunofluorescence (Figure 1B panel 5)

Case 6, (not included in Table 1 since she had a diagnosis of SSc at the initial visit), was a 61-year-old Caucasian female who developed tingling fingertips; carpal tunnel syndrome was suspected. The tingling was followed by swelling and pain in her hands and sclerodermatous skin changes to her forearm. She visited UFCAD and had a diagnosis of diffuse SSc. RP developed two years later.

Two patients (cases 1 and 2), who had anti-RNAP III without SSc but later developed SSc, three patients who did not meet the SSc criteria during observation (cases 3 to 5), and a patient who had SSc without RP and developed RP two years later (case 6), were classified as "atypical presentation" cases. Anti-RNAP III antibodies in these 6 cases were compared with 15 cases of "typical presentation" in which patients had SSc with RP when they visited clinic and serum samples were collected.

Sera from all six cases of "atypical presentation" clearly immunoprecipitated RNAP I and III (Figure 1A). The intensity of RNAP I and III were similar in two patients who developed SSc later (cases 1and 2, lanes 1 and 2 in Figure 1A) and a patient with SSc who developed RP two years after the development of SSc (case 6, lane 6). In contrast, RNAP I was predominant with much weaker RNAP III in three patients, (cases 3 to 5, lanes 3 to 5 in Figure 1A), who did not have a diagnosis of SSc. RNAP III and II are known to distribute in the nuclei while RNAP I localizes to the nucleoli [9,10]. Consistent with their localization patterns, sera from cases 3 to 5 (panels 3 to 5 in Figure 1B) that had predominant RNAP I IP, had dominant nucleolar staining compared with their nuclear staining. Case 5 had ACA in addition (Figure 1B panel 5).

Levels of anti-RNAP III were tested by ELISA comparing cases with atypical presentation vs typical presentation of SSc (Figure 1C). Levels of anti-RNAP III in the former group were lower than those in the latter (*P *= 0.04 by Mann-Whitney test). Also, anti-RNAP III ELISA negative (< 20 units) was common in the former (3 of 6) vs the latter group (1 of 15, *P *= 0.0526 by the Fisher's exact test). Specifically, 2 of the ELISA negative patients were cases 3 and 4 who had week bands by IP and did not develop scleroderma. Thus, although all cases immunoprecipitated RNAP III, levels of anti-RNAP III by ELISA were lower in cases with atypical clinical presentations compared with those in typical SSc.

Sequential sera from cases 2, 4, and 6 were available for testing by anti-RNAP III ELISA. In case 2, levels of anti-RNAP III were high (99 units) at the initial visit despite complete lack of scleroderma or RP, indicating that anti-RNAP III can be produced prior to clinical manifestations similar to other disease marker autoantibodies. Levels of anti-RNAP III went up when the patient developed sclerodermatous skin changes followed by RP. In case 4, anti-RNAP III became positive while the patient was followed up for ILD, but no clinical changes were observed. In case 6, the patient had low levels of anti-RNAP III when she visited UFCAD with diffuse SSc but without RP. Her anti-RNAP III levels increased from 33 units to 107 units to 112 units prior to the development of RP two years later.

RP is often the first symptom of SSc and may start many years prior to development of SSc [2]. Since cases of anti-RNAP III positive patients who developed sclerodermatous changes prior to RP were noted, the sequence of RP and sclerodermatous changes were reviewed carefully, comparing SSc patients with anti-RNAP III vs other specificities in the UFCAD cohort (Table 2 cases 3 to 5 are not included). Almost all of the SSc patients had RP during the course of the disease regardless of the autoantibody specificity. However, only 3 of 17 anti-RNAP III positive patients did not have RP by the time of initial visit (*P *= 0.07 vs topo I group, *P *= 0.01 vs all others combined). When the medical history was carefully reviewed, sclerodermatous changes appeared prior to RP in 31% (5 of 16) of anti-RNAP III patients while this occurred only in one anti-topo I positive patient in other groups (RNAP III vs topo I, *P *= 0.03; RNAP III vs ACA, *P *= 0.04; RNAP III vs others, *P *= 0.002). The development of RP and sclerodactyly were separated by more than one year only in 25% of anti-RNAP III patients vs 50 to 58% of individuals with other specificities (*P *= 0.08 vs all others). The time between RP and the development of scleroderma was shorter in anti-RNAP III vs ACA group or all others combined (*P *= 0.03 by Mann-Whitney).

**Table 2:** Raynaud's phenomenon and autoantibodies in scleroderma patients

	RNAP III (*n *= 18)	Topo I (*n *= 24)	ACA (*n *= 15)	U3RNP (*n *= 9)	Th/To (*n *= 8)
Prevalence of RP	94% (16/17)	96% (23/24)	100% (15/15)	100% (9/9)	87% (7/8)
Absence of RP at first visit (in RP positive cases)	18% (3/16)^1, 2^	0% (0/23)^2^	0% (0/15)	0% (0/9)	0% (0/7)
Scleroderma prior to RP	31% (5/16)^3, 4, 5^	4% (1/23)^3^	0% (0/15)^4^	0% (0/9)	0% (0/8)
RP to scleroderma > 1 y	25% (4/16) ^6^	53% (10/19)	58% (7/12)	50% (4/8)	57% (4/7)
RP to scleroderma (year, mean ± SD)	1.5 ± 5.3^7, 8^ (0.2 ± 1.2)^9^	4.1 ± 8.9	5.7 ± 5.8^7^	1.1 ± 1.7	2.6 ± 3.4

RP, Raynaud's phenomenon

^1 ^RNAP III vs others, *P *= 0.01; ^2 ^RNAP III vs Topo I, *P *= 0.07; ^3 ^RNAP III vs Topo I, *P *= 0.03; ^4 ^RNAP III vs ACA, *P *= 0.04; ^5 ^RNAP III vs others, *P *= 0.002; ^6 ^RNAP III vs others, *P *= 0.08; ^7 ^RNAP III vs ACA, *P *= 0.03; ^8 ^RNAP III vs others, *P *= 0.03; ^9 ^Value after excluding an outlier that has 21 years interval between RP to scleroderma.

^1-6 ^by Fisher's exact test, ^7,8 ^by Mann-Whitney

## Discussion

Anti-RNAP III antibodies are considered highly specific for the diagnosis of SSc. Among five patients who did not meet the SSc criteria at the initial visit, two developed SSc during follow-up; however, three did not (Table 1). Case 3 had RP and an episode consistent with scleroderma renal crisis and Ccase 4 had ILD. Although their SSc-like features involving internal organs are limited compared with reported cases [11,12], it seems reasonable to suspect that they had a pathogenetic condition similar to systemic sclerosis *sine *scleroderma, in particular with detection of anti-RNAP III. Similar cases of scleroderma renal crisis with minimal or no features of SSc, some with anti-RNAP III antibodies, have been reported [13,14]. Although we should not classify all ANA-positive acute renal failure or ILD as systemic sclerosis *sine *scleroderma, identifying SSc-specific autoantibodies may prove useful in understanding the pathogenetic mechanism and selecting treatment options.

Detection of anti-RNAP III by ELISA in patients with diagnosis other than SSc was occasionally reported, but most of them were interpreted as false positives based on negative results by IP [15,16]. The presence of anti-RNAP III confirmed by IP in non-SSc patients, as shown in the present study, was rarely reported [4,15]. Several studies compared ELISA and IP to estimate specificity and sensitivity of ELISA. IP positive ELISA negative (false negative) is reported in 4 to 9% [15-17], while IP negative ELISA positive (false positive) is 12 to 15% [15,16]. Although confirmation of IP negative appears to be incomplete, other study suggests that false positive by ELISA is as low as ~2% [17]. False positives appear to be more common among weakly positive samples or in non-SSc patients [15,16]. Our data showed that 19% (4 of 21, 1 later became positive) of IP positives were ELISA negative (Figure 1). Anti-RNAP III ELISA has been shown to have a good sensitivity and specificity [15-17] and has made the testing for this common SSc antibody widely available. Thus, it significantly helps clinical practice since IP is not available to most clinicians. Nevertheless, it should be kept in mind that there are false positives and false negatives in ELISA. In particular, cautious interpretation will be necessary for weakly positive samples or positives among non-SSc patients. IP should remain the gold standard for anti-RNAP III antibody testing.

It is of interest that all three cases of non-SSc anti-RNAP III positive patients had predominant RNAP I reactivity with weak RNAP III reactivity and had a strong nucleolar staining that is not always seen in anti-RNAP I/III positive SSc patients [17,18]. In addition, anti-RNAP III levels increased prior to development of scleroderma or RP in cases 2 and 6, suggesting a correlation between levels of anti-RNAP III and disease activity. One study suggested a link between increasing levels of anti-RNAP III after the initial visit and increasing total skin score and onset of renal crisis over time [15]. Another study reported an association of anti-RNAP III levels and skin score and a negative correlation with a pulmonary function test [19]. These are consistent with the present cases; however, the course of anti-RNAP III during the onset of sclerodermatous skin changes or RP has not been reported previously.

A unique feature observed in anti-RNAP III positive SSc patients was the late development of RP, which has also been suggested [2], but details were not reported. Even if RP appears prior to scleroderma, the interval is often within a year, consistent with the previously reported rapidly progressive nature of SSc in anti-RNAP III positive patients [2,20]. The development of RP after the initial visit was observed in only three cases with anti-RNAP III but did not appear in other groups at all, and 31% (5 of 16) in the anti-RNAP III group had scleroderma prior to RP. In contrast, RP preceded scleroderma in most cases of SSc [2]. Sclerodermatous skin without RP is considered characteristic of malignancy-associated pseudoscleroderma [21-23]. However, anti-RNAP III positive SSc should be considered in the differential diagnosis of scleroderma without RP, since 31% of our anti-RNAP III positive SSc developed sclerodermatous changes prior to RP.

## Conclusions

In summary, anti-RNAP III is highly specific for SSc and related conditions even in an unselected population from a rheumatology clinic. In cases with atypical SSc, dominance of anti-RNAP I and strong nucleolar staining may be seen. The unusual presentation of the occurrence of scleroderma without RP appears to be characteristic of anti-RNAP III positive SSc. In some cases, internal organ involvement, such as renal or lung disease, may precede skin manifestation of SSc, and detection of anti-RNAP III provides useful diagnostic and prognostic information.

## Abbreviations

ACA: anticentromere antibodies; ACR: American College of Rheumatology; ANA: antinuclear antibodies; GAVE: gastric antral vascular ectasia; ILD: interstitial lung disease; IP: immunoprecipitation; IRB: Institutional Review Board; MCPs: metacarpophalangeal joints; PIPs: proximal interphalangeal joints; PH: pulmonary hypertension; PM/DM: polymyositis/dermatomyositis; RNAP: ribonucleic acid polymerase; RF: rheumatoid factor; RP: Raynaud's phenomenon; SLE: systemic lupus erythematosus; SS: Sjögren's syndrome; SSc: systemic sclerosis: scleroderma; Topo I: topoisomerase I; UFCAD: University of Florida Center for Autoimmune Diseases

## Competing interests

RWB and TTW are employees of INOVA Diagnostics. All other authors have no competing interests.

## Authors' contributions

MEK, YL, SJR, JYFC, EKLC, RWB, TTW and MS carried out the immunoassays. AC and MS designed the study. MS performed the statistical analysis. MRB, ESS and WHR enrolled patients for the study and maintained the database. AC, MS and EKLC drafted the manuscript. All authors read and approved the final manuscript.
